# Clinical characteristics, management, and prognosis of pembrolizumab-induced immune-related oral mucositis

**DOI:** 10.3389/fimmu.2026.1710588

**Published:** 2026-01-28

**Authors:** Liuxian Yu, Jun Li, Yaxi Tang

**Affiliations:** Department of Stomatology, The First Affiliated Hospital of Hunan Traditional Chinese Medical College (Hunan Provincial Directly Affiliated Hospital of Traditional Chinese Medicine), Zhuzhou, China

**Keywords:** immune-related adverse event, management, mucous membrane pemphigoid, oral mucositis, pembrolizumab

## Abstract

**Background:**

Pembrolizumab-induced immune-related oral mucositis (irOM) is a rare and often underrecognized toxicity. This study aimed to systematically characterize its clinical profile, histopathologic patterns, management strategies, and outcomes to support timely diagnosis and evidence-based care.

**Methods:**

A comprehensive search of PubMed, EMBASE, Web of Science, WanFang Data, and CNKI was performed using a combination of MeSH terms (e.g., “Pembrolizumab,” “Oral Mucositis,” “Stomatitis,” “Mucous Membrane Pemphigoid,” and “Immune-Related Adverse Events”) and free-text terms (e.g., “anti-PD-1” and “checkpoint inhibitor toxicity”), with Boolean operators (AND/OR) applied to maximize retrieval; reports published up to July 31, 2025, were included. The quality of case reports was evaluated using the JBI Critical Appraisal Checklist.

**Results:**

Among 18 patients, the median age was 72 years (range 15, 88) and 72.2% were male. The median onset of irOM was 24 weeks (range 3, 66), consistent with a delayed presentation. Clinically, painful oral ulcers or erosions were most frequently observed (50.0%), followed by dysphagia or odynophagia (27.8%). Histopathologic evaluation most often revealed a pemphigoid-like pattern (27.8%) or mixed inflammatory infiltrates (22.2%), with additional findings including ulceration with granulation tissue, lichenoid mucositis, plasma cell infiltrates, and epithelial hyperplasia. Systemic corticosteroids were the mainstay of therapy (88.9%), while pembrolizumab was discontinued in one-third of cases (33.3%). Refractory disease occasionally required immunomodulatory agents such as methotrexate (16.7%) or infliximab (11.1%). Clinical outcomes were generally favorable, with 88.9% of patients achieving symptomatic improvement or remission and a median recovery time of 6 weeks (range 2, 52). On rechallenge, 3 of 4 patients had no recurrence.

**Conclusion:**

Pembrolizumab-induced irOM usually develops after months of treatment, presenting as ulcerative mucositis, sometimes extending to the airway or esophagus. Biopsy may show pemphigoid-like changes. Corticosteroids are effective, and immunosuppressants can be used for refractory cases. Recovery is typically within weeks and rechallenge is feasible for select patients.

## Introduction

Immune-related oral mucositis (irOM) is a common complication of immune checkpoint inhibitor therapy, particularly in patients treated with programmed cell death 1 (PD-1) inhibitors (1, 2). Clinically, irOM presents with painful ulcerations, erosions, and mucosal inflammation in the oral cavity, which can severely affect the patient’s ability to eat and speak, thus compromising their quality of life. In some cases, the condition may extend into the larynx or esophagus, leading to odynophagia and dysphagia (3). These oral manifestations are often associated with other immune-related adverse events (irAEs) such as cutaneous and gastrointestinal toxicities, but they remain relatively undercharacterized in the literature compared to other mucocutaneous toxicities (4).

The pathogenesis of irOM is thought to involve T-cell disinhibition and humoral immune activation, which collectively drive epithelial injury and inflammation in the mucosal layers of the oral cavity (5, 6). Immune-mediated damage to the epithelial cells leads to ulcerative mucositis, lichenoid interface mucositis, and even pemphigoid-like changes, characterized by subepithelial clefting and basement membrane zone immunoreactivity on direct immunofluorescence (7). This complex immune response underscores the necessity for careful diagnostic evaluation, including early biopsy and endoscopy, to differentiate irOM from other conditions such as infections, radiation-related mucositis, and autoimmune bullous diseases (8).

Pembrolizumab, a PD-1 inhibitor, is one of the most widely used immunotherapy agents in clinical practice and has revolutionized the treatment of various malignancies. Emerging reports suggest that pembrolizumab-induced irOM may manifest with heterogeneous phenotypes and variable time to onset, creating diagnostic uncertainty and management delays that can compromise nutrition, infection risk, and oncologic continuity of care (2). Current treatment approaches are largely based on general guidelines for managing immune-related adverse events: corticosteroids are the mainstay of treatment, while for refractory cases, a combination treatment strategy tailored to the patient’s individual circumstances may be required (9). Decisions on treatment interruption, continuation, or rechallenge remain individualized, and prospective evidence is lacking.

Given these gaps, a focused review on pembrolizumab-related irOM is crucial to guide clinical practice. This study aims to systematically synthesize the available data to define the clinical profile, endoscopic and biopsy signatures, therapeutic approaches, outcomes including recovery time, and recurrence risk upon rechallenge. Our goal is to provide evidence-based recommendations that support early recognition and optimized management of pembrolizumab-induced irOM in clinical settings.

## Methods

### Study design and search strategy

We performed a retrospective, case-based synthesis of pembrolizumab-associated irOM. A systematic search of PubMed, EMBASE, Web of Science, WanFang Data, and CNKI identified reports published up to July 31, 2025. The search strategy incorporated both controlled vocabulary (MeSH terms) and free-text terms. Specifically, we used MeSH terms such as “Pembrolizumab,” “Oral Mucositis,” “Stomatitis,” “Mucous Membrane Pemphigoid,” and “Immune-Related Adverse Events,” in combination with free-text terms like “anti-PD-1” and “checkpoint inhibitor toxicity.” The search strategy also included Boolean operators such as AND and OR to ensure comprehensive retrieval of relevant studies. Reference lists of eligible articles were hand-searched to capture additional cases.

### Inclusion and exclusion criteria

We included case reports or case series that attributed oral mucositis to pembrolizumab and provided sufficient patient-level information for extraction. Reports were excluded if they were reviews, mechanistic or non-human studies, conference abstracts without extractable data, duplicate publications, or lacked adequate clinical details.

### Study selection and data extraction

Two reviewers independently screened titles/abstracts and full texts, with disagreements resolved by a third reviewer. A piloted extraction form captured demographics, cancer type, pembrolizumab regimen (dose, schedule, cycles), time to mucositis onset, clinical manifestations, endoscopic and histopathologic findings, management of irOM (topical and systemic therapies, adjunct immunomodulators), immune checkpoint inhibitor management (continuation, hold, discontinuation), rechallenge and recurrence, clinical outcomes, and recovery time. When multiple publications described the same patient, the most complete dataset was retained.

### Quality assessment of case reports

The methodological quality of the included case reports and case series was evaluated using the Joanna Briggs Institute (JBI) Critical Appraisal Checklist for Case Reports, which covers eight domains (https://jbi.global/critical-appraisal-tools). Two reviewers independently rated each domain as “Yes,” “No,” “Unclear,” or “Not applicable,” and discrepancies were resolved through discussion with a third reviewer.

### Causality assessment

Drug–event relatedness was graded using the WHO–Uppsala Monitoring Centre criteria as “certain,” “probable,” “possible,” or “unlikely,” integrating temporal association, rechallenge, alternative explanations, and rechallenge information when available.

### Statistical analysis

Variables were summarized descriptively. Categorical data are presented as counts and percentages. Continuous data are reported as medians with ranges (minimum to maximum). Analyses were performed with SPSS version 23.0.

## Results

### Basic characteristics

**Figure 1:**
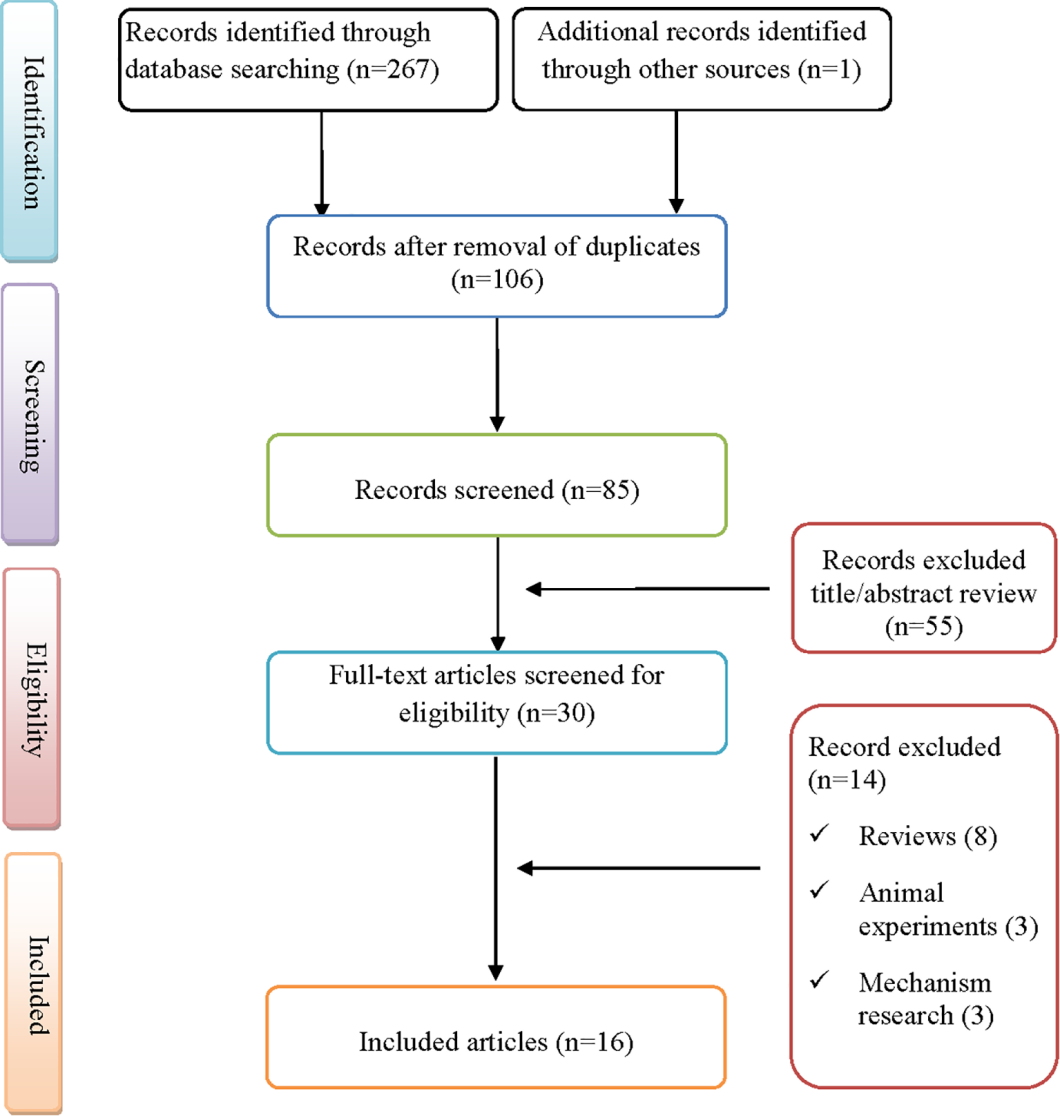
Flowchart illustrating the study selection process for inclusion.

As illustrated in **Figure 1**, a total of 267 records were retrieved through database searches and manual screening. After removal of duplicates and assessment of titles and abstracts, 16 studies were eligible for final inclusion (10–25). These studies collectively reported 18 patients with pembrolizumab-associated irOM, which formed the basis of the present analysis (**Supplementary Table 1**). Overall, reporting quality was generally adequate across the 8 JBI domains, with most cases providing clear patient demographics, clinical presentation, diagnostic workup, interventions, outcomes, and key clinical lessons. Item-level ratings for each case are summarized in **Supplementary Table 2**.

As shown in **Table 1**, the median age was 72 years (range 15, 88), with a male predominance (72.2%). Cases were reported across ten countries, most commonly the United States (38.9%), with additional reports from Canada (11.1%), Israel (11.1%), Chile (5.6%), China (5.6%), France (5.6%), Germany (5.6%), Korea (5.6%), India (5.6%), and Brazil (5.6%). Underlying malignancies in the included cases were heterogeneous. lung cancer was most frequent (27.8%), followed by head and neck squamous cell carcinoma (11.1%), urothelial carcinoma (11.1%), and melanoma (11.1%), with single cases each of renal cell carcinoma, ovarian clear cell adenocarcinoma, endometrial carcinoma, anaplastic astrocytoma, and undifferentiated pleomorphic sarcoma. The median time from pembrolizumab initiation to irOM onset was 24 weeks (range 3, 66), indicating a predominantly delayed presentation; notably, 27.8% occurred between 51–66 weeks. Among patients with dosage information, the typical regimen was 200 mg every 3 weeks. Concomitant therapies were infrequently reported and included axitinib, lenvatinib, or radiation.

**Table 1:** Basic information of 18 patients with pembrolizumab-induced immune-related oral mucositis.

Parameter	Classification	Value
Gender (18)^a^	Male	13 (72.2%)
	Female	5 (27.8%)
Age (18)^a^	Years	72 (15, 88)^b^
Country (18)^a^	United States	7 (38.9%)
	Canada	2 (11.1%)
	Israel	2 (11.1%)
	Chile	1 (5.6%)
	China	1 (5.6%)
	France	1 (5.6%)
	Germany	1 (5.6%)
	Korea	1 (5.6%)
	India	1 (5.6%)
	Brazil	1 (5.6%)
Symptom onset time (18)^a^	Weeks	24 (3, 66)^b^
	1-10	4 (22.2%)
	11-20	3 (16.7%)
	21-30	3 (16.7%)
	31-40	1 (5.6%)
	41-50	2 (11.1%)
	51-66	5 (27.8%)
Indication (18)^a^	Lung cancer	5 (27.8%)
	Head and neck squamous cell carcinoma	2 (11.1%)
	Urothelial carcinoma	2 (11.1%)
	Melanoma	2 (11.1%)
	Renal cell carcinoma	1 (5.6%)
	Merkel cell carcinoma	1 (5.6%)
	Ovarian clear cell adenocarcinoma	1 (5.6%)
	Endometrial carcinoma	1 (5.6%)
	Anaplastic astrocytoma	1 (5.6%)
	Undifferentiated pleomorphic sarcoma	1 (5.6%)
Pembrolizumab dosage (11)^a^	200 mg every 3 weeks	7 (63.6%)
	100 mg every 3 weeks	1 (9.1%)
	2 mg/kg every 3 weeks	3 (2.73%)
Concomitant medications (3)^a^	Axitinib	1 (33.3%)
	Lenvatinib	1 (33.3%)
	Radiation	1 (33.3%)

a Represents the number of patients with this parameter out of 18 patients.

b Median (minimum, maximum).

### Clinical manifestations

Among the 18 patients (**Table 2**), the most frequently observed presentation was painful oral ulcers or erosions, reported in 50.0% of cases. Dysphagia or odynophagia was noted in 27.8%. Extension to the upper aerodigestive tract was described in several patients, with laryngeal involvement in 16.7% and esophageal involvement in 11.1%. Other manifestations included gingival lesions in 11.1%, extra-oral mucosal involvement such as nasal, conjunctival, pharyngeal, or cutaneous lesions resembling Stevens-Johnson syndrome in 16.7%, and systemic symptoms such as weight loss in 5.6%.

**Table 2:** Clinical information of 18 patients with pembrolizumab-induced immune-related oral mucositis.

Parameter	Classification	Value
Clinical symptoms (18)^a^	Painful oral ulcers/erosions	9 (50.0%)
	Dysphagia/odynophagia	5 (27.8%)
	Laryngeal involvement (hoarseness, airway edema/obstruction)	3 (16.7%)
	Esophageal involvement (esophagitis, mucositis extension)	2 (11.1%)
	Gingival disease (gingivitis, gum pain, enlargement)	2 (11.1%)
	Extra-oral mucosal lesions (nasal, conjunctival, pharyngeal, skin/SJS)	3 (16.7%)
	Weight loss/systemic manifestations	1 (5.6%)
Endoscopic/Biopsy (16)^a^ findings	Pemphigoid pattern	6 (37.5%)
	Inflammatory infiltrate (lymphocytic/ neutrophilic/eosinophilic)	4 (25.0%)
	Ulceration/granulation tissue	3 (18.8%)
	Lichenoid mucositis/interface pattern	1 (6.3%)
	Plasmacytosis/polyclonal plasma cell infiltrate	1 (6.3%)
	Epithelial hyperplasia/parakeratosis/mild atypia	1 (6.3%)

a Represents the number of patients with this parameter out of 18 patients.

### Endoscopic and histopathologic findings

Endoscopic examinations typically revealed mucosal ulcerations, erosions, or areas of diffuse erythema involving the oral cavity, gingiva, and occasionally extending into the pharynx, larynx, or esophagus. Some cases demonstrated airway edema or narrowing, highlighting the potential risk of upper aerodigestive tract compromise. Biopsy findings were diverse with recurring patterns. The most common was a pemphigoid-like change with subepithelial clefting and linear IgG/C3 along the basement membrane zone (27.8%). Mixed inflammatory infiltrates of lymphocytes, plasma cells, or neutrophils were present in 22.2% of cases. Additional features included ulceration with granulation tissue, lichenoid interface mucositis, epithelial hyperplasia, and plasma cell rich mucositis. Taken together, these features indicate that pembrolizumab-induced irOM can resemble autoimmune bullous or lichenoid disease and highlight the importance of early biopsy with direct immunofluorescence for accurate differentiation.

### Treatment and outcome

As summarized in **Table 3**, systemic corticosteroids were the mainstay of treatment and were administered in 88.9% of cases. Pembrolizumab was discontinued in 33.3%, while the remainder either continued therapy or temporarily held dosing based on clinical severity. For patients with refractory disease, additional immunomodulators were introduced, including methotrexate (16.7%) and infliximab (11.1%). Topical agents, supportive care, and antifungals or antibiotics were occasionally prescribed as adjunctive measures. Clinical outcomes were generally favorable. Overall, 88.9% of patients experienced symptomatic improvement or complete remission. The median recovery time among those with available data was 6 weeks (range 2, 52). Despite improvement in most cases, delayed healing or non-recovery was noted in a minority. Four patients underwent pembrolizumab rechallenge, and three tolerated re-exposure without recurrence. According to the WHO-UMC causality assessment, most cases were categorized as probable (13/18; 72.2%), and the remainder as possible (5/18; 27.8%). These findings highlight a strong temporal association and treatment response in most reports, while underlining the need for careful causality evaluation in complex clinical contexts.

**Table 3:** Treatment and prognosis of 18 patients with pembrolizumab-induced immune-related oral mucositis.

Parameter	Classification	Value
Treatment (18)^a^	Discontinuation	6 (33.3%)
	Corticosteroids	16 (88.9%)
	Topical anesthetics/mouth rinses	3 (16.7%)
	Doxycycline	2 (11.1%)
	Infliximab	2 (11.1%)
	Methotrexate	3 (16.7%)
	Opioid analgesics	1 (5.6%)
	Vitamin supplementation (folate/B12)	1 (5.6%)
	Tacrolimus	1 (5.6%)
	Rituximab	1 (5.6%)
	Mycophenolate mofetil	1 (5.6%)
	Opioid analgesics	1 (5.6%)
Outcome (18)^a^	Complete remission	3 (16.7%)
	Improvement	13 (72.2%)
	No recovery	1 (5.6%)
	Death (cancer progression related)	1 (5.6%)
Recovery time (15)^a^	Weeks	6 (2, 52)^b^
Rechallenge (4)^a^	No recurrence	3 (75.0%)
	Unspecified	1 (25.0%)
WHO-UMC causality category (18)^c^	Possible	5 (27.8%)
	Probable	13 (72.2%)

a Represents the number of cases describing this parameter out of 18 patients.

b Median (minimum, maximum).

c According to the WHO-UMC criteria, a “certain” classification requires a clear temporal association with drug exposure, resolution after discontinuation, and recurrence on rechallenge. A “probable” classification is assigned when the time course is compatible, alternative causes are unlikely, and improvement occurs after drug withdrawal without rechallenge. A “possible” classification applies when the temporal link is reasonable but other explanations remain plausible and the effect of withdrawal is inconclusive.

## Discussion

Pembrolizumab-induced irOM is rare but clinically significant, representing an underrecognized subset of mucocutaneous immune-related adverse events (irAEs) (13). Although dermatologic toxicities such as rash and pruritus are relatively common, oral involvement is seldom reported, which contributes to diagnostic delays and inappropriate empirical antifungal or antibacterial treatment (26). By synthesizing 18 patient-level cases from 16 published reports, our analysis provides a more comprehensive view of this toxicity, delineating its clinical spectrum, latency, pathology, management, and outcomes. The median onset of irOM across cases was 24 weeks after treatment initiation, which is later than the typical cutaneous manifestations observed with checkpoint inhibitors. Pembrolizumab has a long terminal half-life of approximately 25 days and achieves sustained PD-1 receptor occupancy, which may contribute to the delayed onset of mucosal toxicities even after drug discontinuation. This finding is consistent with individual case reports describing delayed presentations, sometimes appearing after prolonged pembrolizumab exposure or even after therapy discontinuation. Such latency reinforces the importance of long-term vigilance for mucosal toxicities throughout the treatment course and during follow-up. Clinically, ulcerative mucositis was the predominant feature, often associated with severe oral pain, impaired oral intake, and weight loss. Importantly, several cases involved the larynx or esophagus, resulting in airway edema, dysphonia, aspiration risk. These findings highlight the need for multidisciplinary evaluation, including otolaryngology or gastroenterology, when patients present with dysphagia, odynophagia, or airway symptoms. Histopathologic findings across the reviewed cases were variable. The most frequent pattern was pemphigoid-like change with subepithelial clefting and linear IgG/C3 deposition, occasionally accompanied by circulating BP180 antibodies. Other reported features included plasma cell-rich infiltrates, ulceration with granulation tissue, and lichenoid interface mucositis. This diversity underscores the importance of biopsy with direct immunofluorescence for accurate diagnosis and for distinguishing pemphigoid-spectrum disease from non-bullous mucositis.

The underlying mechanisms remain incompletely defined. PD-1/PD-L1 signaling normally contributes to peripheral tolerance by regulating T-cell and B-cell responses (8, 27). Its blockade can unleash autoreactive immunity targeting epithelial adhesion molecules, producing mucosal blistering and ulceration (28). The identification of anti-BP180 antibodies in some cases supports a humoral autoimmune process, whereas the steroid-responsiveness of ulcerative mucositis without antibody evidence points to a T-cell–driven interface injury (29). The coexistence of both mechanisms across cases suggests overlapping immune pathways with variable dominance in individual patients (30). Potential risk modifiers, such as prior radiotherapy, combination systemic therapy, or baseline autoantibody status, have been hypothesized but remain unproven (8, 31).

Management strategies across the literature were largely consistent with general irAE guidelines (32, 33). Systemic corticosteroids were the cornerstone of treatment, with most patients showing symptomatic improvement or remission within weeks. Adjunctive measures included topical rinses, anesthetics, and vitamin supplementation. However, a subset of refractory cases required escalation to steroid-sparing immunosuppressants, including methotrexate, infliximab, rituximab, or mycophenolate mofetil. These interventions were particularly valuable in pemphigoid-like cases with multisite mucosal involvement or risk of scarring. The majority of patients improved, but delayed recovery and persistence of symptoms were reported in isolated cases. Importantly, rechallenge with pembrolizumab did not uniformly result in recurrence: three of four rechallenged patients tolerated re-exposure without relapse, whereas others in the literature experienced recurrence, highlighting the individualized nature of risk-benefit decisions.

Diagnostic challenges remain an important theme. In many cases, oral candidiasis was initially suspected, and patients were treated empirically with antifungals without benefit. Only after persistence of symptoms and subsequent biopsy was irOM recognized and appropriately managed with corticosteroids. Similarly, distinguishing immune-related mucositis from radiation-induced toxicity or drug-related lichenoid reactions is particularly difficult in patients with head and neck cancers. A structured diagnostic approach that integrates timely biopsy, microbiological exclusion, and early initiation of immunosuppressive therapy once alternative causes are excluded appears essential to avoid prolonged morbidity.

Future directions should include prospective registries and collaborative efforts to define the incidence, risk factors, and optimal therapeutic approaches for irOM. Comparative studies of steroid-sparing immunomodulators in refractory disease are especially warranted, as is the exploration of biomarkers such as autoantibody profiles or mucosal cytokine signatures that may predict susceptibility or recurrence. Until such data become available, clinicians should maintain vigilance for delayed oral toxicities in pembrolizumab-treated patients, ensure early biopsy with immunopathology, initiate corticosteroid therapy once infection and tumor progression are excluded, and make individualized decisions regarding immunotherapy continuation or rechallenge in a multidisciplinary context.

### Limitations of the study

This study has several limitations due to its reliance on case reports from the existing literature. Firstly, it is based on case reports and small series, which are susceptible to publication bias, incomplete data, and heterogeneity in the reporting of clinical course, histology, and treatment. Secondly, the quality of case reports and missing data limit our ability to conduct a comprehensive quantitative evaluation. Furthermore, there are currently no RCTs, cohort studies, or case-control studies specifically addressing pembrolizumab-induced immune-related oral mucositis (irOM), which further impacts the generalizability of our findings. Future research could provide opportunities for quantitative synthesis or meta-analysis once more studies are published. Nonetheless, by pooling available cases across diverse tumor types and geographic regions, this analysis identifies reproducible clinical patterns and highlights the importance of multidisciplinary recognition and management.

## Conclusion

Pembrolizumab-induced irOM is uncommon but clinically important, typically presenting after months of therapy with painful ulcerations and occasional laryngeal or esophageal involvement. Diagnosis relies on clinical assessment supported by biopsy and direct immunofluorescence rather than imaging. Corticosteroids are the mainstay of treatment, adjunct immunomodulators are useful for refractory disease, and most patients recover within weeks; decisions on interruption or rechallenge should be individualized. Further studies should define incidence and risk factors, standardize diagnostic criteria, evaluate steroid-sparing strategies, and establish evidence-based guidance for safe rechallenge.

## Data Availability

The original contributions presented in the study are included in the article/**Supplementary Material**. Further inquiries can be directed to the corresponding author.
